# The Gut-brain Connection and Episodic Migraine: an Update

**DOI:** 10.1007/s11916-023-01175-6

**Published:** 2023-10-04

**Authors:** Linda Nguyen, Nada Hindiyeh, Sutapa Ray, Robert E. Vann, Sheena K. Aurora

**Affiliations:** 1 Stanford University, Palo Alto, CA USA; 2 Metrodora Institute, West Valley City, UT USA; 3 Impel Pharmaceuticals, Seattle, WA USA

**Keywords:** Gut-brain, Migraine, Episodic migraine

## Abstract

**Purpose of review:**

Historical evidence suggests a shared underlying etiology for migraine and gastrointestinal (GI) disorders that involves the gut-brain axis. Here we provide narrative review of recent literature on the gut-brain connection and migraine to emphasize the importance of tailoring treatment plans for patients with episodic migraine who experience GI comorbidities and symptoms.

**Recent findings:**

Recent population-based studies report the prevalence of migraine and GI disorders as comorbidities as well as overlapping symptomology. American Headache Society (AHS) guidelines have integrated GI symptoms as part of migraine diagnostic criteria and recommend nonoral therapies for patients with GI symptoms or conditions. Nasal delivery is a recommended nonoral alternative; however, it is important to understand potential adverse events that may cause or worsen GI symptoms in some patients due to the site of drug deposition within the nasal cavity with some nasal therapies. Lastly, clinical perspectives emphasize the importance of identifying GI symptoms and comorbidities in patients with episodic migraine to best individualize migraine management.

**Summary:**

Support for an association between the gut-brain axis and migraine continues to prevail in recent literature; however, the relationship remains complex and not well elucidated. The presence of GI comorbidities and symptoms must be carefully considered when making treatment decisions for patients with episodic migraine.

## Introduction

The bidirectional communication between the central nervous system and gastrointestinal (GI) system is referred to as the gut-brain axis and has been implicated in the pathogenesis of several neurological conditions [1••, 2••, 3]. As such, the Rome Foundation adopted the term *disorder of gut-brain interaction* (DGBI), defined as a group of disorders classified by GI symptoms related to any combination of motility disturbances, visceral hypersensitivity, altered mucosal and immune function, gut microbiota, and central nervous system processing. DGBIs were previously referred to as functional GI disorders. Historically, these disorders have been characterized by their symptoms and were without known structural abnormalities. Examples of DGBIs are functional dyspepsia, cyclic vomiting syndrome (CVS), irritable bowel syndrome (IBS), functional constipation, and functional diarrhea [4]. Migraine is a debilitating headache disorder with a high prevalence and burden, and like DGBIs, migraine is more prevalent in women than men [5–9]. According to the Global Burden of Disease Study in 2019, migraine is the second among the world’s causes of disability and the top cause for young women aged 15–49 years [8]. The International Classification of Headache Disorders version 3 (ICHD-3) defines migraine as a recurrent headache disorder with moderate or severe headache attacks lasting 4 to 72 h that are accompanied by nausea and photophobia and/or phonophobia. Individuals can further experience chronic migraine (CM), defined as ≥ 15 headaches per month, or episodic migraine (EM), defined as ≤ 14 headaches per month [5, 10]. In addition to nausea, individuals with migraine routinely experience other GI symptoms, including vomiting, diarrhea, and constipation and GI comorbidities such as celiac disease, gastroesophageal reflux disease (GERD), IBS, *Helicobacter pylori* infection, gastroparesis, functional dyspepsia, and CVS [11–19]. Further, several therapies are indicated to treat both migraine and accompanying symptoms and GI disorders, which include metoclopramide, tricyclic antidepressants (TCAs), antiepileptic drugs, antiemetics, and noninvasive vagal nerve stimulation [20, 21•, 22–26]. These associations suggest a relationship between migraine and the GI system, implicating pathophysiology of the gut-brain axis. Recent studies demonstrate a highly complex interplay between multiple physiological systems to produce the broad range of symptomology observed with migraine and DGBI or GI disorders. Several comprehensive review papers have been published on the topic of the gut-brain axis and its relationship to migraine [1••, 27••, 28–31]. Here, we provide a narrative review of current evidence for an association between migraine and GI comorbidities and symptoms, with an emphasis on episodic migraine; explore the effects of EM treatments on those symptoms based on recent evidence from the literature; and make treatment recommendations to optimize outcomes in patients with EM who experience GI comorbidities and symptoms.

## Evidence Linking Migraine and GI Disorders

Historically, a shared pathophysiology between migraine and GI disorders has been based on the observed overlap of their symptom profiles and prevalence studies demonstrating a comorbid relationship. For example, individuals with migraine can experience nausea, vomiting, and delayed gastric emptying, which also constitute part of the symptomology of many well-known GI disorders, such as gastroparesis, functional dyspepsia, and CVS [4, 5, 12, 32]. Autonomic dysfunction has been described in migraine and GI disorders and may account for overlapping symptoms [33–39]. According to a prospective, cross-sectional study of 605 participants with migraine, visceral autonomic symptoms are common in individuals with migraine in all phases of the migraine cycle. Results showed that 41% of participants reported that 100% of their migraine attacks were accompanied by autonomic symptoms. Visceral symptoms present before, during, and after a migraine attack included stomach fullness, bloating, nausea, vomiting, eructing, constipation, diarrhea, frequent defecation, and frequent urination, and occurred more frequently during a migraine attack. Participants with migraine and ≥ 1 visceral symptom during their migraine attack reported a longer duration for their migraine compared with participants without visceral symptoms (24.4 ± 29.6 h vs 16.8 ± 19.8 h; p = 0.008). Participants with visceral symptoms also experienced migraine attacks of greater severity based on the numeric rating scale-11 compared with participants without visceral symptoms both before (8 ± 1.7 vs 7.6 ± 1.8; p = 0.02) and during (8.01 ± 1.7 vs 7.09 ± 1.9; p = 0.001) a migraine attack [37]. A prospective longitudinal study in 43 participants from a single tertiary academic center who were diagnosed with episodic or CM used the Composite Autonomic Symptom Scale (COMPASS-31) questionnaire to determine the presence of autonomic symptoms, which sums the scores of 6 domains: orthostatic intolerance (range 0–40), vasomotor (range 0–5), secretomotor (range 0–15), GI (range 0–25), bladder (range 0–10), and pupillomotor (range 0–5). A total score of 0 indicates no presence of autonomic symptoms and a score of 100 represents severe autonomic symptoms. Autonomic symptoms were reported in this cohort of patients with migraine and no change in the COMPASS-31 score before and after treatment over 12 months was observed (30.3 vs 30.3, respectively; p = 0.885), suggesting there is no correlation between treatment response and severity of autonomic symptoms [38].

Emerging evidence suggests an association between altered gut microbiota and migraine pathophysiology. A cross-sectional, case control study that evaluated gut microbiota in 42 participants with EM demonstrated altered gut microbiota when compared with 43 healthy controls. Moreover, the relative abundance of specific microbial genera was shown to be associated with migraine frequency and severity [40]. Altered gut microbiota have also been noted in individuals with fibromyalgia and IBS [41, 42], suggesting an association between gut microbiota, pain, and proinflammatory mediators. In support of this notion, a recent preclinical study demonstrated that microbiota dysbiosis enhanced migraine-like pain via upregulation of the proinflammatory cytokine tumor necrosis factor alpha (TNFα) in intraspinal trigeminal nucleus caudalis (Sp5C) [43]. A randomized, double-blind, placebo-controlled trial of 50 chronic and 50 episodic patients with migraine in Iran evaluated migraine treatment effect of supplementation with a 14-strain probiotic mixture over 8 and 10 weeks, respectively. In patients with EM who received probiotics compared with those who received placebo, the mean frequency of migraine attacks per month significantly decreased (-2.64 vs 0.06; respectively, p < 0.001) and the mean migraine severity as assessed by the visual analog scale decreased (-2.14 vs 0.11, respectively; p < 0.001). In patients with CM who received probiotics compared with those who received placebo, the mean frequency of migraine attacks significantly decreased (-9.67 vs -0.22; respectively, p ≤ 0.001) and the mean migraine severity decreased (-2.69 vs -0.22, respectively; p ≤ 0.001). The number of abortive drugs used weekly for EM and daily for CM also decreased compared with baseline (EM: -0.72; p < 0.001; CM: -1.02; p < 0.001) and with no significant change in the placebo group [44]. However, a recent meta-analysis of published randomized clinical trials found insufficient evidence that probiotic supplementation significantly affects migraine frequency or severity, indicating that probiotics alone may be insufficient to restore microbiota alterations associated with migraine pathogenesis and that further research is warranted in this field [45].

Several recent population-based studies have reported the prevalence of migraine and GI disorders as comorbidities; however, most of these studies do not disclose if migraine is chronic or episodic. For example, in the large Migraine in America Symptoms and Treatment (MAST) study (n = 6045), 75.7% of participants with migraine reported experiencing nausea, and 28.1% described nausea as their most bothersome symptom [46]. In a separate analysis of the MAST cohort (n = 15,133), participants with migraine were more than 3 times likely to experience gastric ulcers and GI bleeding than healthy controls (OR: 3.11; 95% CI: 2.8–3.5) [47]. In a study of 60 patients with functional dyspepsia, of whom 38 had postprandial distress syndrome and 22 had epigastric pain syndrome, 68% (41/60) were reported to have migraine without aura. Of those with postprandial distress syndrome and epigastric pain syndrome, 76% (29/38) and 54% (12/22) had migraine without aura, respectively. In patients with postprandial distress syndrome who had migraine, onset was related to meal ingestion and associated with dyspeptic symptoms for 89%, and there was a statistically significant correlation of migraine severity and postprandial modification of the gastric discomfort threshold (r = -0.73; p < 0.001) [13]. A cross-sectional Iranian study of overweight and obese individuals with migraine evaluated their prevalence of GI disorders, which was 18.8% for dyspepsia, 11% for constipation, 7.2% for heartburn, 6.1% for fatty liver, 4.4% for IBS, and 1.7% for cholelithiasis [48]. A retrospective analysis in the United States showed a high prevalence of headaches (48%) in participants with IBS (n = 1645) [49]. A cross-sectional, observational study of 341 Iranian participants undergoing gastric endoscopy demonstrated that 43.7% met diagnostic criteria for migraine, with a statistically significant higher incidence of women (67.8%) compared with men (32.2%; p = 0.003). Among the participants with GERD, *H pylori* infection, duodenal ulcer, and gastric ulcer, 78, 138, 37, and 10 participants had migraine, respectively, which was statistically significant (p ≤ 0.001) compared with participants who did not have migraine for all GI disorders with the exception of gastric ulcer [50]. The American Migraine Prevalence and Prevention Study of 11,603 eligible respondents with migraine showed that 7.4%, 10.2%, and 10.2% of patients with low-frequency (n = 7860; 0–3 monthly headache days), medium-frequency (n = 2051; 4–7 monthly headache days), and high-frequency (n = 898; 8–14 monthly headache days) EM, respectively, experienced ulcers of the stomach or intestines, which was statistically significant for high- vs medium-frequency EM (p = 0.026) [51]. A Japanese cross-sectional study of individuals with EM (n = 271) reported comorbidities of heartburn (38.4%), chronic constipation (27.3%), frequent diarrhea (23.6%), IBS (16.6%), GERD (15.9%), ulcers (10.3%), and ulcerative colitis (1.5%) [52]. Lastly, a multicenter, randomized, single-blind, phase 4 study in 65 patients with EM without significant GI symptoms evaluated colonic transit time of a single dose of a calcitonin gene-related peptide (CGRP) monoclonal antibody that targets the ligand (galcanezumab; Emgality®, Eli Lilly, Indianapolis, IN, USA) compared with a CGRP monoclonal antibody that targets the receptor (erenumab; Aimovig®, Amgen Inc, Thousand Oaks, CA, USA). Baseline demographic data revealed that gastric emptying time was severely delayed in 12.5% and 21.2%, small bowel transit time was delayed in 28.1% and 18.2%, colonic transit time was delayed in 12.5% and 12.1%, small and large bowel transit time was delayed in 12.5% and 12.1%, whole gut transit time was delayed in 15.6% and 15.2%, the Bristol Stool Form Scale was consistent with constipation in 9.4% and 9.1%, and the Bristol Stool Form Scale was consistent with diarrhea in 3.1% and 9.1% of patients in the erenumab and galcanezumab groups, respectively. This growing body of literature highlights the need for careful examination of migraine and any related GI symptoms or comorbidities, which in turn will serve to optimize EM treatment regimens and improve patient outcomes.

## The Impact of GI Symptoms and Disorders on EM Treatment

EM can be managed with acute therapies to reduce the pain, associated symptoms, and disability or preventive therapies if a patient meets the appropriate diagnostic requirements [21•, 53, 54]. According to the American Headache Society (AHS) consensus, the main goals of acute therapies for migraine are rapid and consistent freedom from pain and associated symptoms without recurrence, restored ability to function, minimal need for repeat dosing or rescue medications, optimal self-care, reduced subsequent use of resources, minimal or no adverse events (AEs), and cost considerations [21•]. For migraine preventive therapies, the main goals are to reduce attack frequency, severity, duration, and disability; improve responsiveness to and avoid escalation in use of acute treatment; improve function; reduce disability; reduce reliance on poorly tolerated, ineffective, or unwanted acute treatments; reduce overall cost associated with migraine treatment; enable patients to manage their disease to enhance a sense of control; improve health-related quality of life; and reduce headache-related distress and psychological symptoms [21•]. Approved acute therapies can be divided into migraine-specific agents, such as triptans, ergotamine derivatives (eg, dihydroergotamine [DHE] mesylate), gepants, and ditans, and migraine-nonspecific agents, such as nonsteroidal anti-inflammatory drugs (NSAIDs) and combination nonopioid analgesics. Approved preventive therapies for EM include the migraine-specific agents of CGRP monoclonal antibodies and migraine-nonspecific agents of beta-blockers, anticonvulsants, and antidepressants. Dual therapies for migraine can be used as both acute and preventive treatments, which include frovatriptan (for menstrual-related migraine), neuromodulation, behavioral therapy, and gepants (such as rimegepant) [21•]. Based on the consensus from several international guidelines as well as results from clinical trials, the aforementioned acute and preventive therapies are safe and efficacious for the management of migraine [21•, 53, 54], and “a nonoral formulation should be used in patients whose attacks are associated with severe nausea or vomiting, who do not respond well to traditional oral treatments, patients who experience significant nausea or vomiting early during attacks, or who have trouble swallowing orally administered medications” [7, 21•]. Most acute therapies are orally administered, with some available as parenteral or nasal, while preventive therapies are either oral or parenteral [21•, 55]. A strong understanding of how route of administration coupled with mechanism of action of migraine therapies affects treatment outcomes is critical for drug selection and meeting individualized needs.

While oral medications are convenient and easy to administer, their absorption and efficacy may be compromised in patients with GI symptoms or comorbidities. As early as the 1990s, studies have demonstrated that gastric emptying in patients with migraine is delayed during and outside the migraine attack [12, 56–59]. Complementing these results are early studies reporting slower absorption of orally administered drugs, including some triptans, during a migraine attack, which was shown to be increased when combined with agents that improve GI motility [60–66]. Further, nausea and vomiting are highly disabling symptoms accompanying migraine that may interfere with the efficacy or administration of oral therapies [46, 67–69]. A prospective cohort study using data from the American Migraine Prevalence and Prevention Study reported that 43.7% of participants with EM (n = 3182) experienced persistent frequent nausea and 3.4% progressed to CM within 2 years, indicating the importance of considering nausea during drug selection for managing EM in patients [70]. Although triptans are generally recommended as first-line acute therapies for moderate to severe migraine attacks, a recent retrospective claims analysis in the United States of 10,509 new triptan users reported that 30% were potential triptan-insufficient responders, with 68% of potential triptan-insufficient responders using opioids in the 24-month postindex period. This highlights the need for better, individualized acute treatment options that are nonoral [71]. An absence of baseline vomiting and nausea has been found to be a predictor of headache relief at 2 h with oral sumatriptan 100 mg using data from the Sumatriptan Naratriptan Aggregate Patient database, and in some studies, triptan use was reported to be the cause of nausea [72–75]. A post hoc analysis of the randomized, double-blind COMPASS study that compared AVP-825 (Onzetra® Xsail®; breath-powered intranasal delivery of powdered sumatriptan 22 mg) with oral sumatriptan tablets (100 mg) characterized the longitudinal trajectories of nausea across multiple migraine attacks using 3 models. The first model (n = 259) measured overall nausea (ie, longitudinal change in nausea from predose to 120 min for the whole sample, independent of baseline nausea). Compared to oral sumatriptan, overall nausea declined more rapidly during the first hour, and there were reduced odds of nausea from 30 to 120 min following AVP-825 treatment. The second model (n = 232) measured treatment-emergent nausea from 10 to 120 min postdose in migraine attacks without nausea at baseline. Compared to AVP-825, the risk of treatment-emergent nausea increased at a significantly faster rate over 45 min postdose, with significantly greater odds of treatment-emergent nausea at 45, 60, and 90 min postdose with oral sumatriptan. The third model (n = 167) measured nausea relief from 10 to 120 min postdose in eligible migraine attacks with nausea at baseline, and it showed reduced odds of nausea with AVP-825 treatment compared with oral sumatriptan but no differences in the rate of change in nausea over time between the 2 treatments [76]. A separate post hoc analysis of the COMPASS trial of 259 patients further revealed that AVP-825 significantly lowered mean pain intensity and mean disability from 10 to 90 min postdose (effect sizes: -0.09 to -0.29; p < 0.0001 to 0.01) and was associated with greater within-person consistency in migraine pain intensity and migraine-related disability across multiple migraine attacks, from 45 to 120 min postdose compared with oral sumatriptan [77]. Results from these studies suggest that nasal administration may provide more rapid and consistent drug absorption and provide better relief from nausea compared with oral administration.

Overall, nasal drug delivery can provide rapid onset of relief, bypasses first-pass metabolism, and improves drug bioavailability in a noninvasive and convenient manner [78–85], which is particularly important for patients with GI symptoms who have cycled through many oral agents without treatment success. However, the site of drug deposition within the nasal cavity needs to be considered because it can influence drug pharmacokinetics [79, 81, 86, 87]. Traditional nasal sprays generally deliver drug to the lower nasal space, where epithelium is not well suited for optimal drug absorption and where there is an increased likelihood of drug clearance due to nasal drip, swallowing, or mucociliary clearance, potentially resulting in variable absorption and suboptimal efficacy as well as AEs of dysgeusia [79, 83, 86–93]. According to the Food and Drug Administration Adverse Event Reporting System (FAERS) database, dysgeusia is a common adverse event of nasal sprays, which is most likely a result of the taste of the drug rather than the underlying mechanism of action of the therapies [94, 95]. Several factors may contribute to the incidence of dysgeusia associated with nasal sprays for migraine treatment, such as type of medication used, dosage, and individual characteristics. Traditional nasal sprays that use a nasal spray pump or an atomizer for delivery of the drug have shown to deposit drug in the lower nasal space [79, 83, 86, 93, 96], and dysgeusia may be caused by postnasal dripping of residual spray into the oral cavity and contact with the posterior tongue and taste buds, which activates the taste receptor cells [95, 97]. Regardless of the mechanism, dysgeusia is a concern for patients with migraine who experience GI symptoms because it can exacerbate existing nausea and vomiting and, in some cases, be the cause of it [95]. Dysgeusia rates for approved nasally administered acute therapies for migraine range from 1.1% to 25%, with the lowest value referring to DHE mesylate delivered to the upper nasal space by Precision Olfactory Delivery (POD®) or INP104 (TRUDHESA®, Impel Pharmaceuticals, Seattle, WA, USA) [84, 98–104]. INP104 is approved as an acute treatment for migraine with or without aura [103]. POD is a handheld and manually actuated device that gently delivers a narrow, focused plume of DHE mesylate through the nasal valve, reaching the hard-to-reach upper nasal space, where drug is less likely to drip out of the nose or be swallowed and is more likely to be absorbed within the richly vascularized olfactory epithelium [96]. The site of drug deposition and the POD technology are likely explanations for the low rates of dysgeusia (1.1%) in the phase 3 study of INP104 in patients with migraine [84]. Parenteral administration of migraine drugs is another alternative to oral agents and can provide rapid onset of relief; however, it may not be ideal for all patients. For example, intravenous DHE mesylate can produce strong adverse events of nausea and vomiting due to high maximum plasma concentrations and must be delivered in a clinical setting with an antiemetic, making it inconvenient for routine administration [83, 87, 105–107]. Further, parenteral administration is not ideal for patients who are needle-phobic.

The CGRP class of acute and preventive therapies, which act via antagonism of the CGRP pathway by targeting the CGRP receptor or its canonical receptor, or the CGRP molecule itself, have proven clinical efficacy and safety [21•, 108]. A recent narrative review provides evidence for the role of CGRP in migraine and GI disorders [109••]. Based on the FAERS database, GI-related AEs such as abdominal discomfort and pain, diarrhea, nausea, constipation, and vomiting have been reported with CGRP monoclonal antibody use. Constipation rates were higher for CGRP receptor antagonists compared with antagonists that target the CGRP ligand [109••], which may be due to differences in mechanisms of action [110]. Results from the phase 4 study in 65 patients with EM without significant GI symptoms that evaluated colonic transit time of a single dose of a CGRP monoclonal antibody that targets the ligand (galcanezumab) compared with a CGRP monoclonal antibody that targets the receptor (erenumab) demonstrated numerically reduced colonic transit times for galcanezumab and numerically increased colonic transit times for erenumab at 2 weeks post-treatment but were not statistically significant. Participants treated with erenumab experienced both a reduction in the number of spontaneous bowel movements and a hardening of stool at 2 and 4 weeks following treatment, whereas participants treated with galcanezumab showed no change in these measures. Notably, participants receiving both treatments reported significant increases in the constipation domain of the GI symptom rating scale [110]. This result is consistent with another study of 30 healthy participants in Denmark, in which 93% treated with infusions of CGRP experienced GI symptoms, including rumbling, nausea, diarrhea, and urge to defecate. Further, symptoms of GI hyperactivity were noted at the time of peak CGRP plasma concentration [111]. Taken together, these studies emphasize that additional care with respect to GI adverse events—especially constipation—should be taken into consideration for drug selection in patients with migraine and GI symptoms.

## Migraine and Gut Disorders: a Clinical Perspective

**Fig. 1:**
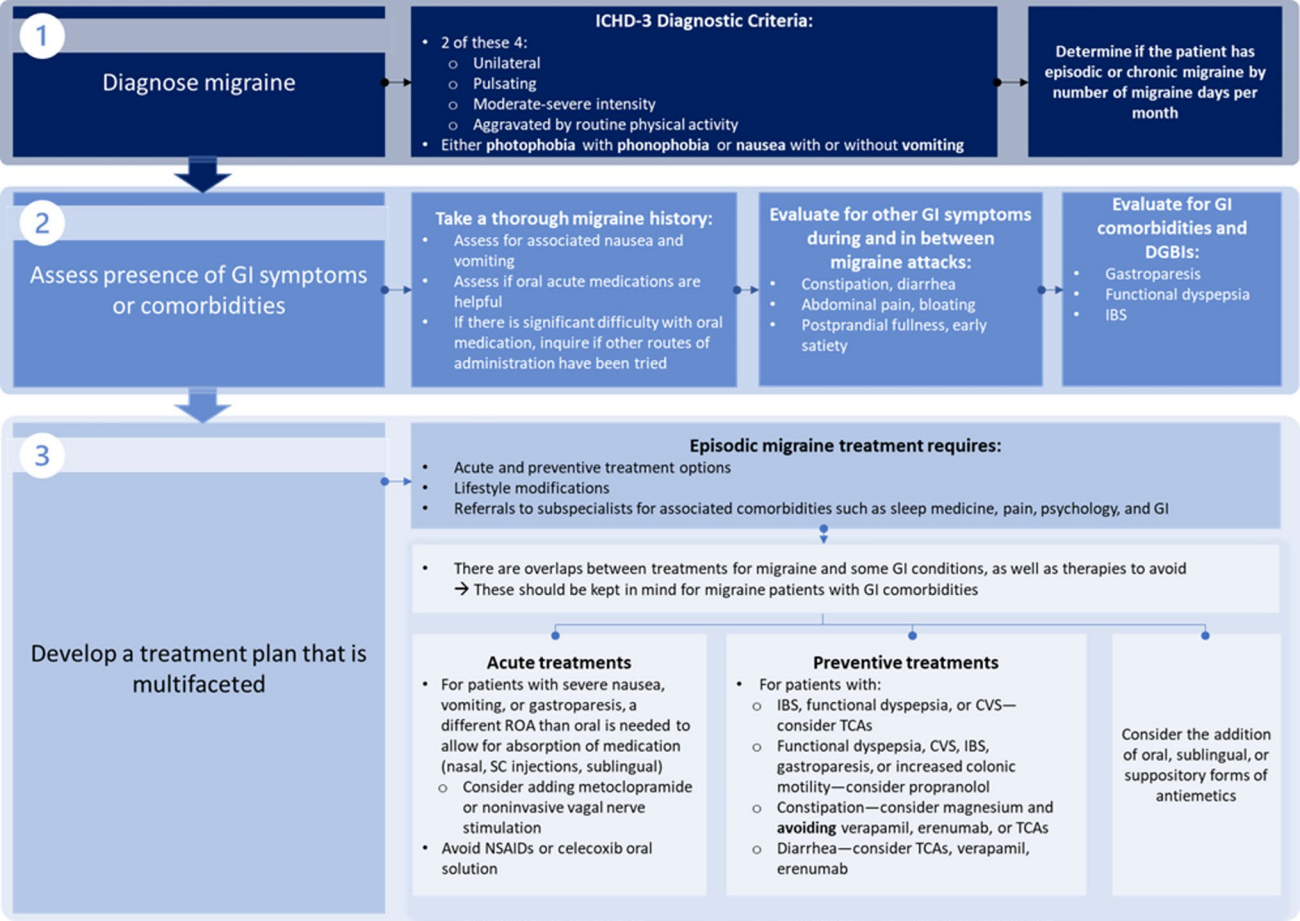
Proposed Step-by-step Algorithm for Diagnosing GI Symptoms or Comorbidities and Developing a Treatment Plan for Patients With Episodic Migraine [5, 21•]. Note: This figure was created based on clinical perspective of the authors as well as published guidelines from the American Headache Society Consensus Statement. CVS, cyclic vomiting syndrome; DGBI, disorder of gutbrain interaction; GI, gastrointestinal; IBS, irritable bowel syndrome; ICHD-3, International Classification of Headache Disorders version 3; NSAID, nonsteroidal anti-inflammatory drug; ROA, route of administration; SC, subcutaneous; TCA, tricyclic antidepressant

A growing body of literature highlights the importance of detecting GI comorbidities and symptoms in patients with migraine when considering available treatment options; however, established guidelines in the headache community are lacking. Migraine requires a multifaceted treatment approach because of its complex pathophysiology and broad array of potential comorbidities. An optimal treatment plan for EM requires acute and preventive therapeutic options, lifestyle modifications, and referrals to subspecialists for associated comorbidities, such as sleep medicine, pain, psychiatry, and GI. Referrals to GI specialists should be especially considered when patients are significantly bothered by GI symptoms and their migraine medications are not effective. To determine the presence of GI comorbidities or symptoms, it is recommended that clinicians take a thorough migraine history and inquire whether oral, acute medications are helpful. If the patient mentions difficulties with oral medications, question whether other routes of migraine drug administration have been attempted. Clinicians should also inquire about the presence of GI symptoms during and between migraine attacks, including constipation, diarrhea, abdominal pain, bloating, postprandial fullness, and early satiety. The choice of acute and preventive treatment options for EM should heavily consider the presence of and type of GI symptoms or comorbidities. For example, acute treatments for patients with severe nausea or vomiting or gastroparesis requires a different route of administration than oral to allow for absorption of medication. Metoclopramide can also be used for gastroparesis or to accelerate gastric emptying. Similarly, TCAs should be considered as a preventive migraine treatment for a patient with CVS or functional dyspepsia; however, if a patient experiences frequent constipation, then TCAs are not recommended (Fig. 1). According to the AHS consensus, nonoral alternatives include subcutaneous and intranasal sumatriptan, intranasal and intramuscular ketorolac, subcutaneous and intranasal DHE mesylate, and neuromodulatory devices. Some acute therapies for migraine may cause GI-related adverse events, and the benefit-risk profiles of these agents should be weighed carefully in patients with EM and GI symptoms or comorbidities. NSAIDs are a recommended acute treatment for migraine, but they can cause serious GI adverse events. Celecoxib oral solution should be avoided in patients with a history of peptic ulcer disease or GI bleeding because of an increased risk of spontaneous bleeding, ulceration, and perforation of the stomach or intestines. Further, alternatives to erenumab are recommended in patients with constipation [21•].

## Conclusions

The association between the gut-brain axis and migraine has recently garnered considerable interest in the headache community and attention to GI comorbidities, and symptoms may be particularly important in managing patients with EM who do not experience relief from oral therapies or who cycle through many migraine therapies with suboptimal efficacy or adverse events. Other considerations include route of drug administration and formulation, which can affect absorption, and therefore the efficacy and tolerability of migraine therapies. Using a multifaceted treatment approach, understanding which migraine therapies to consider or avoid in patients with GI symptoms or comorbidities, and knowing when to refer to GI specialists can help patients with EM to achieve their individualized needs and optimize their migraine management.
